# Cardiopulmonary Exercise Testing in Repaired Tetralogy of Fallot: Multiparametric Overview and Correlation with Cardiac Magnetic Resonance and Physical Activity Level

**DOI:** 10.3390/jcdd9010026

**Published:** 2022-01-13

**Authors:** Benedetta Leonardi, Federica Gentili, Marco Alfonso Perrone, Fabrizio Sollazzo, Lucia Cocomello, Stefani Silva Kikina, Rachel M. Wald, Vincenzo Palmieri, Aurelio Secinaro, Maria Giulia Gagliardi, Attilio Parisi, Attilio Turchetta, Lorenzo Galletti, Massimiliano Bianco, Fabrizio Drago

**Affiliations:** 1 Department of Cardiology and Cardiac Surgery, Bambino Gesù Children’s Hospital IRCCS, 00165 Rome, Italy; benedetta.leonardi@opbg.net (B.L.); mgiulia.gagliardi@opbg.net (M.G.G.); attilio.turchetta@opbg.net (A.T.); lorenzo.galletti@opbg.net (L.G.); fabrizio.drago@opbg.net (F.D.); 2 Department of Sport Medicine, Bambino Gesù Children’s Hospital IRCCS, 00165 Rome, Italy; federica.gentili@opbg.net; 3 Department of Cardiology and University Sports Centre, University of Rome Tor Vergata, 00133 Rome, Italy; 4 Unità Operativa Complessa di Medicina dello Sport e Rieducazione Funzionale, Fondazione Policlinico Universitario Agostino Gemelli IRCCS, Università Cattolica del Sacro Cuore, 00168 Rome, Italy; fabriziosollazzo.md@gmail.com (F.S.); stefanisilva.kikina01@icatt.it (S.S.K.); vincenzo.palmieri@unicatt.it (V.P.); massimiliano.bianco@policlinicogemelli.it (M.B.); 5 Bristol Heart Institute, University of Bristol, Bristol BS2 8HW, UK; nn18747@bristol.ac.uk; 6 Peter Munk Cardiac Centre and Joint Department of Medical Imaging, University Health Network, University of Toronto, Toronto, ON M5S 1A1, Canada; rachel.wald@uhn.ca; 7 Advanced Cardiothoracic Imaging Unit, Department of Imaging, Bambino Gesù Children’s Hospital IRCCS, 00165 Rome, Italy; aurelio.secinaro@opbg.net; 8 Department of Movement, Human and Health Science, University of Rome Foro Italico, 00135 Rome, Italy; attilio.parisi@uniroma4.it

**Keywords:** tetralogy of Fallot, cardiopulmonary exercise testing, physical activity, cardiac magnetic resonance, exercise capacity, pulmonary valve

## Abstract

Patients with repaired Tetralogy of Fallot (rToF) typically report having preserved subjective exercise tolerance. Chronic pulmonary regurgitation (PR) with varying degrees of right ventricular (RV) dilation as assessed by cardiac magnetic resonance imaging (MRI) is prevalent in rToF and may contribute to clinical compromise. Cardiopulmonary exercise testing (CPET) provides an objective assessment of functional capacity, and the International Physical Activity Questionnaire (IPAQ) can provide additional data on physical activity (PA) achieved. Our aim was to assess the association between CPET values, IPAQ measures, and MRI parameters. All rToF patients who had both an MRI and CPET performed within one year between March 2019 and June 2021 were selected. Clinical data were extracted from electronic records (including demographic, surgical history, New York Heart Association (NYHA) functional class, QRS duration, arrhythmia, MRI parameters, and CPET data). PA level, based on the IPAQ, was assessed at the time of CPET. Eighty-four patients (22.8 ± 8.4 years) showed a reduction in exercise capacity (median peak VO_2_ 30 mL/kg/min (range 25–33); median percent predicted peak VO_2_ 68% (range 61–78)). Peak VO_2_, correlated with biventricular stroke volumes (RVSV: β = 6.11 (95%CI, 2.38 to 9.85), *p* = 0.002; LVSV: β = 15.69 (95% CI 10.16 to 21.21), *p* < 0.0001) and LVEDVi (β = 8.74 (95%CI, 0.66 to 16.83), *p* = 0.04) on multivariate analysis adjusted for age, gender, and PA level. Other parameters which correlated with stroke volumes included oxygen uptake efficiency slope (OUES) (RVSV: β = 6.88 (95%CI, 1.93 to 11.84), *p* = 0.008; LVSV: β = 17.86 (95% CI 10.31 to 25.42), *p* < 0.0001) and peak O_2_ pulse (RVSV: β = 0.03 (95%CI, 0.01 to 0.05), *p* = 0.007; LVSV: β = 0.08 (95% CI 0.05 to 0.11), *p* < 0.0001). On multivariate analysis adjusted for age and gender, PA level correlated significantly with peak VO_2_/kg (β = 0.02, 95% CI 0.003 to 0.04; *p* = 0.019). We observed a reduction in objective exercise tolerance in rToF patients. Biventricular stroke volumes and LVEDVi were associated with peak VO_2_ irrespective of RV size. OUES and peak O_2_ pulse were also associated with biventricular stroke volumes. While PA level was associated with peak VO_2_, the incremental value of this parameter should be the focus of future studies.

## 1. Introduction

Tetralogy of Fallot (ToF) is the most common form of cyanotic congenital heart disease (CHD) and accounts for 10% of all forms of CHD [1,2,3]. Surgical repair of Tetralogy of Fallot (rToF) has significantly improved long-term survival. However, this repair can result in pulmonary regurgitation (PR) and right ventricular (RV) volume overload, which has been associated with RV dilation, RV dysfunction, symptomatic heart failure, ventricular arrhythmia and sudden death [4,5,6,7,8]. In contrast, other patients can have predominantly pressure overload due to residual right ventricular outflow tract obstruction (RVOTO) [9]. Consequently, the exercise capacity of many patients can worsen over time following subjective assessment [10], despite reporting preserved subjective exercise tolerance.

Cardiopulmonary exercise testing (CPET) has been demonstrated to be a valuable tool in the objective assessment of exercise tolerance by measuring the peak oxygen consumption (peak VO_2_) [11,12]. Thus, it has been advocated as a diagnostic and prognostic tool to recognize early signs of clinical deterioration in most CHD patients [12], including the rToF population [13,14,15]. Most patients report no limitations in day-to-day activities. Therefore, in asymptomatic patients, CPET could give objective information about the function of the heart, lungs, and muscles and be a useful tool for helping to choose the optimal timing for pulmonary valve replacement (PVR) in the presence of significant RV dilation with or without dysfunction. Babu-Narayan et al. showed that in rToF patients undergoing PVR, preoperative oxygen uptake was indeed predictive of early postoperative mortality [16]. However, the relationship between RV dilatation and/or dysfunction and some CPET parameters is still poorly understood [17,18]. In addition, its potential usefulness as a tool for risk stratification and patient selection in asymptomatic adolescents and adults with rToF remains unclear. CPET parameters can be affected by age, gender, body size, and physical activity, especially in children and young adults [19,20,21]. In fact, a borderline peak VO_2_ measurement may be difficult to interpret in sedentary patients and may not be a reflection of the underlying cardiorespiratory disease [22,23]. Within this framework, an accurate estimation of the level of physical activity is also essential in rToF patients to define its impact not only on exercise capacity but also on other CPET parameters like ventilatory efficiency (VE/VCO_2_ slope) and oxygen uptake efficiency slope (OUES). These parameters have recently emerged [21], in addition to oxygen consumption, but the clinical utility of these values in the rToF population remains uncertain. Therefore, our aims were to (1) explore the relationship between CPET and MRI parameters in our cohort of rToF patients in order to understand if the RV dilatation and/or dysfunction could affect their functional capacity; (2) assess the VE/VCO_2_ slope and OUES to determine the incremental value of these measurements in the rToF population; (3) identify the mean level of physical activity in the rToF population and consequently explore the correlation between physical activity level and CPET parameters.

## 2. Materials and Methods

### 2.1. Patient Population

We performed a retrospective review of all collaborative rToF patients older than 12 years who underwent both MRI and CPET at Bambino Gesù Children’s Hospital, Rome, between March 2019 and June 2021. We selected 84 patients who performed contemporary CMR and CPET (within 12 months) and did not present a significant decline in clinical status (NYHA functional class). We excluded patients with a previous PVR and patients with residual shunts. Finally, we included the last clinical evaluation of each patient with the record of all adverse clinical events. All-cause mortality, aborted cardiac arrest, documented ventricular fibrillation (VF), sustained ventricular tachycardia (VT) lasting 30 s or longer, atrial arrhythmias (atrial fibrillation, atrial flutter, supraventricular tachycardia), non-sustained VT (NSVT), and/or pacemaker implantation were recorded at follow-up. RV hypertension was evaluated by the tricuspid regurgitation gradient using continuous-wave Doppler ultrasound by echocardiography. Precisely, we measured the velocity of the regurgitant jet and calculated the peak pressure gradient between the right ventricle and right atrium by applying the Bernoulli equation. The study was approved by the Ethics Committee of the Bambino Gesù Children’s Hospital, IRCCS (Prot. Number 341/2015), and all subjects gave written informed consent. The study was conducted in accordance with the Declaration of Helsinki.

### 2.2. CPET Protocol

Informed consent was obtained from the parents or the patient prior to the test. Spirometry was performed prior to each CPET. All patients performed a symptom-limited CPET on a treadmill, undergoing the incremental Bruce protocol. Breath-by-breath expired gas analysis was performed according to international guidelines and recommendations [24,25]. Predicted normal peakVO_2_ values for adult and pediatric patients were derived from the Hansen and Wasserman [26] and Cooper [27] formulas, respectively. Twelve-lead electrocardiographic monitoring was maintained throughout the test. Blood pressure and pulse oximetry were recorded every 2–3 min and at peak exercise. For the CPET data, we focused on all the parameters which are usually collected and assessed these variables using equations that account for age, gender, body height, body weight, and testing modality [26,27,28].

### 2.3. MRI Imaging

MRI examinations were performed on a 1.5 T scanner (AERA 1.5 T scanner, Siemens, Erlangen, Germany), in accordance with previously published imaging protocols [29]. These included multiple sequences to assess anatomy, cine steady-state free precession sequences for volume and function assessment and phase-contrast imaging to measure flow in the pulmonary valve, aortic valve, and in both pulmonary arteries.

### 2.4. Image Analysis

The acquired data were analyzed offline by a single reader on a separate workstation using a cardiac post-processing software (Viewforum, Philips Medical, Best, The Netherlands, CMR42, Circle Cardiovascular Imaging, Calgary, AB, Canada). Assessment of LV and RV volumes was performed by manual segmentation of the endocardial border of both ventricles on short-axis cine images at end-diastole and end-systole, then calculated using the method of discs [30]. Trabeculations and papillary muscles were considered as part of the blood pool [31]. The ejection fraction (EF) was calculated from the measured volumes. All volumes were indexed to body surface area (BSA), calculated using the formula of DuBois et al. (BSA (m^2^) = 0.007184 × Height (cm) 0.725 × Weight (kg) 0.425) with reference to published normal values [32]. Blood flow was calculated from phase-contrast images using a semiautomatic edge-detection algorithm with operator correction. The regurgitant fraction was calculated as retrograde flow divided by the anterograde flow. Pulmonary regurgitation was considered mild if the regurgitant fraction was <20%, moderate between 20 and 40%, and severe >40% [33]. Pulmonary arteries/RV outflow tract stenosis was diagnosed when two or more of the following MRI criteria were fulfilled (in addition to RV pressure ≥45 mmHg on echocardiography): (1) flow velocity across the RV outflow tract or a branch pulmonary artery ≥3 m/sec; (2) abnormal pulmonary artery size based on BSA-adjusted z-scores for the right pulmonary artery (RPA), and left pulmonary artery (LPA); (3) asymmetric blood flow distribution to lung fields (RPA < 40%; LPA < 20%) [34]. The threshold for systolic dysfunction was RVEF ≤ 51% and LVEF ≤ 55%, based on Kawel-Boehm et al.’s article [32].

### 2.5. Physical Activity Evaluation

Physical activity was assessed through the International Physical Activity Questionnaire (IPAQ) [35], which has already been evaluated in the CHD population [36]; IPAQ was administered to each participant before the CPET. The levels of physical activity were classified as “low/inactive”, “moderate” and “vigorous”.

## 3. Statistical Analysis

The descriptive statistics were expressed as the median and interquartile range (IQR) for continuous variables and as counts and percentages for categorical variables.

Linear regression was carried out to investigate the relationship between CPET parameters and cardiac MRI parameters. Two models were fitted for each outcome: the first model was unadjusted, and the second was adjusted for specified confounders. Linear regression was carried out to investigate the relationship between IPAQ (considered a continuous variable ranging from 0 to 2), CPET, and cardiac MRI parameters. Two models were fitted for each outcome: the first model was unadjusted, and the second model was adjusted for specified confounders. Confounders are defined as any factors that are known or plausible causes of the exposure and outcome. We agreed that the following factors could plausibly influence cardiac MRI parameters and CPET outcomes: sex, age at CPET, and level of physical activity. Sex and age at CPET were considered to be confounders of the IPAQ. The normal distribution and homoscedasticity of residuals were checked with Normal Q–Q and Spread–Location plot, respectively. We also checked if there were cases outside of the Cook’s distance. Complete case analysis was performed. All statistical analyses were performed using the R Statistical Software, version 3.2.3 (R Foundation for Statistical Computing, Vienna, Austria).

## 4. Results

### 4.1. Participant Characteristics

Eighty-four consecutive patients were included in this study (51% male). The transannular patch was the most frequent repair (67.5%). The other types of repair included monocusp valve reconstruction (13.3%), infundibular patching (14.5%), RV to pulmonary artery conduit (3.6%), and finally, valvulotomy (1.1%), which was performed in one patient. The median age at surgery was 9 months (IQR 5–16), whereas the median age at referral to our evaluation was 21.1 years (15.0–30.0). The echocardiographic evaluation revealed RV hypertension in 6 patients and normal RV pressure in the remaining. All demographic features are shown in Table 1.

### 4.2. MRI Results

The main MRI parameters are reported in Table 1. Twenty (24%) patients had an RVEDVi ≥ 140 mL/m^2^ and 5 patients (6.0%) had an RVEDVi > 160 mL/m^2^. Depressed RV systolic dysfunction (RV EF < 51%) was evident in 28 patients (33.3%). Thirty-six patients (43.4%) had low to moderate PR and 47 patients (56.6%) had severe PR. Depressed LVEF (i.e., <55%) was reported in 28 patients (33.3%). In 16 patients (19.0%), the EF of both ventricles was found to be below the cut-off value for normality [30]. However, none of the patients in our cohort had an RVEF < 45% and only 2 patients had an RVEF of 45%. Similarly, only 3 patients had an LVEF < 50%, but none below 46%.

### 4.3. CPET Results

Table 1 shows the CPET results. The median peak work rate reached was the Bruce protocol phase 4 (IQR 4; 4). Overall, the median aerobic capacity was decreased in this population (absolute peak VO_2_/Kg 30 mLO_2_/Kg/min (IQR: 25–33) and peak predicted was 68% (IQR 61–78)). Exercise testing was maximal in most cases (median peak respiratory exchange ratio (RER): 1.09 (IQR 1.02–1.19), peak heart rate (HR) as a percentage of predicted was 88% (IQR 85–92)). Median OUES was 1908 mL/min/l/min (IQR 1665–2538) with a percentage in respect to predicted values of 79% (IQR 68–85). Data regarding the O_2_ pulse trend was available from 68 patients. In 85.3% of patients, a flattening pattern was observed, whereas, in the remaining, a normal increase in O_2_ pulse during exercise was seen in 10.3% and a decline in 4.4% of patients. Ventilatory response to carbon dioxide (VE/VCO_2_) was 27.5 (IQR 25.0–29.9) at anaerobic threshold (AT) and 31.1 (IQR 27.3–34.3) at peak exercise, with an overall normal ventilatory efficiency (VE/VCO_2_ slope of 25.4 (IQR 23.6–28.9) at AT and 28.3 (IQR 25.5–31.5) at respiratory compensation (VCP)). Resting oxygen saturation (SpO_2_) was >95% in the whole population, with only 2 people (2.4%) showing exercise-related desaturation. Among all these parameters, OUES showed a strong positive correlation with peak VO_2_ (r = 0.875, *p* < 0.001).

### 4.4. Adverse Cardiac Events

The episodes of non-sustained ventricular tachycardia (NS-VT) were observed in seven patients (8.3%). They were significantly older (30.2 (IQR 15.3–30.8) vs. 22.1 (IQR 15.2–29.8) years, *p* = 0.014), reached a lower peak HR during the stress test (166.9 (IQR 168–181) vs. 174.9 (IQR 167–182.5) bpm, *p* = 0.045) and had a lower LVEF at MRI (53.6 (IQR 55–60) vs. 57.4% (IQR 54–60), *p* = 0.035). None of these patients had an RVEF or an LVEF lower than 47% and 50% at MRI, respectively.

### 4.5. IPAQ Results

**Figure 1:**
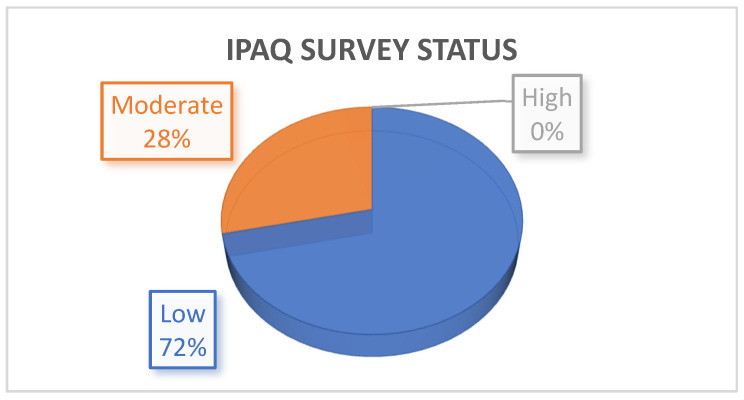
Level of physical activity according to the IPAQ survey.

As shown in Figure 1, none of the participants met the higher level of physical activity. Most of the subjects (58, 71.6%) were classified as “low/inactive”, whereas 23 subjects (28.4%) were considered as “moderate activity” practitioners. Finally, we did not have the IPAQ result of three patients.

### 4.6. Correlations—CPET and MRI

**Figure 2:**
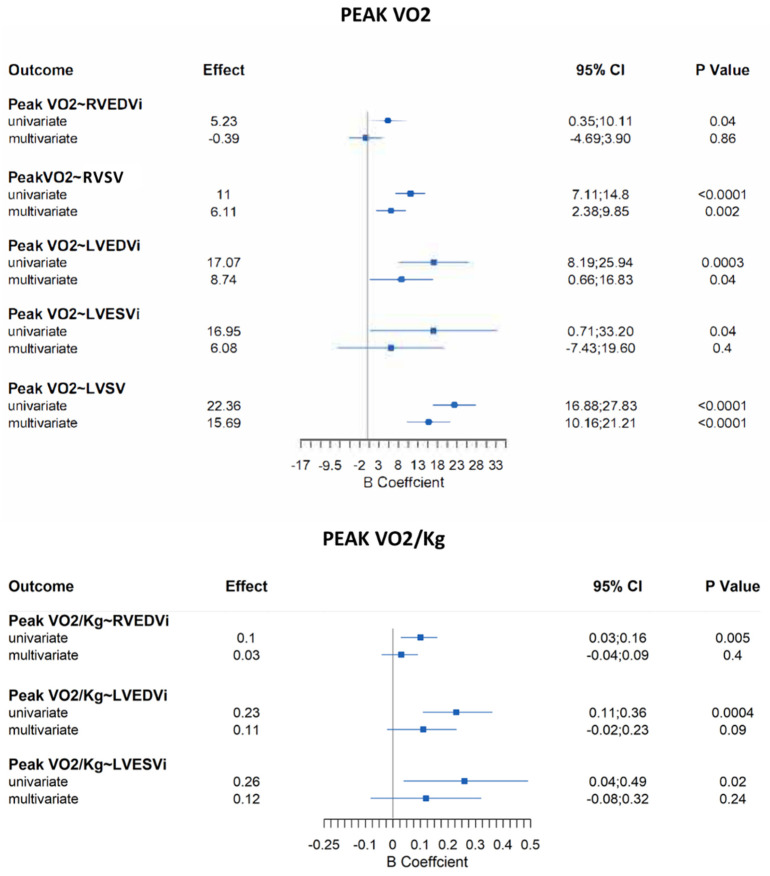
Peak VO_2_ and peak VO_2_/kg coefficients from univariate and confounder adjusted multivariate regression models (only the statistically significant values are reported in this figure. The other results are in the supplemental materials). Legend: LVEDVi: left ventricular end-diastolic volume indexed to body surface area; LVESVi: left ventricular end-systolic volume indexed to body surface area; LVSV: left ventricular stroke volume; RVEDVi: right ventricular end-diastolic volume indexed to body surface area; RVSV: right ventricular stroke volume.

**Figure 3:**
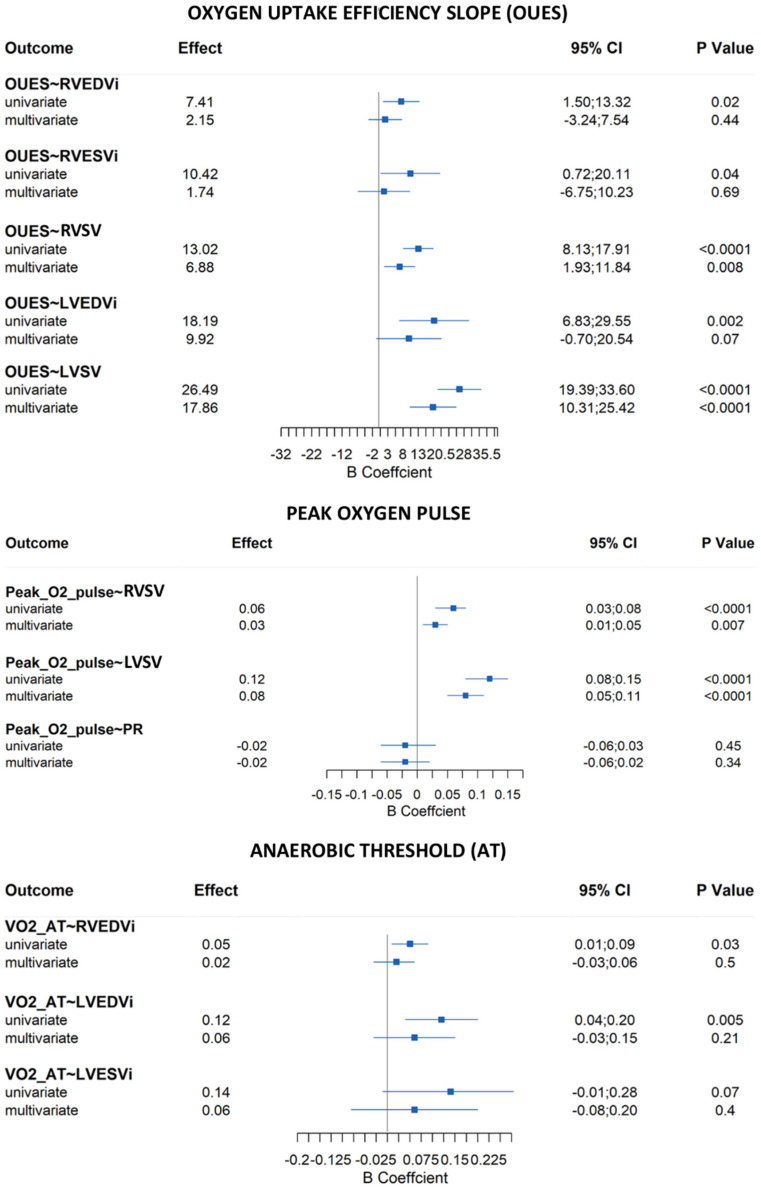
OUES, peak O_2_ pulse and anaerobic threshold coefficients from univariate and confounder adjusted multivariate regression models (only the statistically significant values are reported in this figure. The other figure with the statistically insignificant correlations is reported in the supplemental material). Legend: LVEDVi: left ventricular end-diastolic volume normalized for body surface area; LVESVi: left ventricular end-systolic volume normalized for body surface area; LVSV: left ventricular stroke volume; RVEDVi: right ventricular end-diastolic volume normalized for body surface area; RVESVi: right ventricular end-systolic volume normalized for body surface area; RVSV: right ventricular stroke volume.

On univariate analysis, no correlations were found between CPET parameters and PR, LVEF, or RVEF. RVEDVi was positively correlated with peak VO_2_ (*p* = 0.04), peak VO_2_/kg (*p* = 0.005), VO_2_ at AT (*p* = 0.03), and OUES (*p* = 0.02). RVESVi showed a relationship only with OUES (*p* = 0.04). Interestingly, biventricular stroke volumes were significantly correlated with peak VO_2_, OUES, and peak O_2_ pulse (*p* < 0.05 for all). Finally, LVEDVi, as well as RVEDVi, showed a positive correlation with peak VO_2_, peak VO_2_/kg, VO_2_ at AT, and OUES (*p* < 0.05 for all), whereas LVESVi correlated only with peak VO_2_ (*p* = 0.04) and peak VO_2_/kg (*p* = 0.02). However, on multivariate analysis adjusted for age, gender, and level of physical activity, we only found a significant correlation between biventricular stroke volumes and peak VO_2_, OUES, and peak O_2_ pulse (*p* < 0.05 for all) (Figure 2 and Figure 3). Surprisingly, a relationship between LVEDVi and peak VO_2_ was also seen (Figure 2).

As noted, 28 patients (33.3%) had a reduced RVEF: these patients were older (Table 2) and only showed a statistically significant worse RVESVi in respect to people with a normal RVEF (Table 2). Furthermore, no statistically significant differences were found for any CPET parameter.

### 4.7. Correlations—IPAQ and CPET/MRI

On multivariate analysis adjusted for age and gender, no correlation between the IPAQ and MRI parameters was identified. On the contrary, regarding the CPET parameters, a significant correlation between the IPAQ and peak VO_2_/Kg was highlighted on multivariate analysis (β = 0.02, 95% CI 0.003 to 0.04; *p* = 0.019). On univariate analysis, we documented a statistically significant correlation between the level of physical activity and both peak HR (r 0.25, *p* = 0.011) and the indexes of ventilatory efficiency: VE/VCO_2_ at AT (r 0.25, *p* = 0.015), VE/VCO_2_ slope at AT (r 0.32, *p* = 0.003), and VE/VCO_2_ slope at VCP (r 0.30, *p* = 0.009). On multivariate analysis, no significant correlation between the level of physical activity and the indexes of ventilatory efficiency was found.

## 5. Discussion

### 5.1. Exercise Capacity in the rToF Population and Its Relationship with RV Size and Dysfunction

Our study highlighted a reduction in exercise tolerance in rToF patients, although only 6% of our patients had an RVEF < 47% (none below 45%), 67% had a preserved RVEF (RVEF > 51%) and all had NYHA class I. In particular, a median peak VO_2_/Kg of 30 mL/min and a median percentage of predicted peak VO_2_ of 68% were found, which are the parameters commonly used to document the patient’s cardiovascular status in the rToF population. These data agree with the literature [17,37,38] and, once again, raise the issue of understanding the real state of health of these patients, most of whom report living their lives without any limitations.

The correlation analyses between CPET and cardiac MRI parameters indicated no statistically significant correlation between exercise capacity indexes and PR, RVEF, and LVEF, respectively. These observations are contrary to those previously published [14,17,18]. Among the several associations found in this study, the strongest one concerned RV dimensions, particularly RVEDVi, which positively correlated with peak work capacity and submaximal indexes of exercise capacity on univariate analysis. However, on multivariate analysis adjusted for age, gender, and level of physical activity, the correlation between RVEDVi and CPET parameters did not reach statistical significance. This is probably due to the well-known difference in CPET parameters and ventricular size between males and females and/or between adolescents and adults [11,21]. The only significant correlations found were between biventricular stroke volumes and LVEDVi when adjusted for age, gender, and level of physical activity. Our findings suggest that in a state of hemodynamic compensation, RV dilation leads to an increase in net pulmonary forward flow, LV preload, and LV volume as an attempt to maintain a similar peak aerobic exercise capacity, reinforcing previously published findings [17,39]. Therefore, ToF patients with severe pulmonary insufficiency and RV dilation (within a certain range of RV volumes) are probably able to maintain a satisfactory (albeit not normal) functional capacity within certain limits of the RV dilation and dysfunction [40].

Given our findings that ToF patients with significant pulmonary insufficiency present a clinically acceptable hemodynamic compensation, within certain limits, even those with moderate-severe RV dilatation (i.e., 150 mL/m^2^), it would be interesting to identify the threshold of RV dilation and dysfunction at which the cardiovascular system begins to fail. Hence, future work should be performed including a significantly higher number of subjects, equally divided among those with mild, moderate, and severe dilation (>160 mL/m^2^) and, in the context of dilation, between those with an EF < or >40%. These data could make important changes to the current guidelines on PVR, highlighting new parameters to be taken into consideration within specific values of RV dilation.

Finally, although our population did not include a sufficient number of patients with an RVEF < 51% to be able to accurately assess the impact of RV dysfunction on CPET parameters, we have not observed any significant worsening in the above-mentioned parameters in our group of older patients with an RVEF < 51%. This suggests that the worsening of functional capacity could begin to occur in patients with an RVEF < 40% [18].

### 5.2. Effectiveness of OUES as an Index of Exercise Performance in rToF Patients

Very few data are available on the oxygen uptake efficiency slope (OUES) in the rToF population [21,41], although it has already been demonstrated to be an independent and reproducible measure of cardiorespiratory function that does not require maximal exercise [42]. It has also been proposed to be a valuable prognostic factor in patients with chronic heart failure [43,44], candidates for heart transplant [45] and complex congenital heart disease (Fontan) patients [46]. Our results showed a strong positive correlation between OUES and peak VO_2_ (r = 0.875, *p* < 0.001), thus proposing OUES as an effective submaximal index of exercise performance even for rToF patients. OUES showed a significant correlation with biventricular stroke volumes and LVEDVi on multivariate analysis, reconfirming that OUES should be considered as an additional parameter to evaluate patients with compensated rToF, even more so in the presence of a test that does not reach an RER ≥ 1.10.

### 5.3. Usefulness of the IPAQ Survey in rToF Patients

Above all, despite a subjectively reported preserved exercise tolerance (NHYA class I), very few patients (28.4%) engaged in regular physical activity, and no one in our series reported being engaged in high volumes of physical activity according to the IPAQ survey.

As for the relationship between the IPAQ survey results and both MRI and CPET parameters, we found a significant correlation between the IPAQ and peak VO_2_/Kg on multivariate analysis adjusted for age and gender, reflecting a possible positive effect of even a moderate level of physical activity on exercise capacity in the rToF population. The effectiveness of exercise training on improving peak VO_2_/Kg in cardiovascular diseases is currently well documented, even in CHD [47,48,49,50,51] yet there has been little discussion on prescribing regular physical activity to ToF patients to improve their long-term prognosis.

We believe the significant correlation between the IPAQ and peak VO_2_/Kg observed in our study to be an important result because self-reported methods of assessment such as the IPAQ are cheap and can be easily incorporated into routine clinical practice [52,53,54,55,56,57] allowing us to obtain additional data about the regular lives of rToF patients. The integration of the data obtained from CPET with the real level of physical activity of these patients based on their IPAQ score could allow us to better understand the clinical status of rToF patients, highlighting even small initial decreases in myocardial reserve in apparently asymptomatic patients. Therefore, future studies on a larger population are needed to better understand the real impact of a given level of physical activity on VO_2_ values in ToF patients, knowing their degree of RV dilation and/or dysfunction. That is to say, a certain VO_2_ value that is considered normal for a sedentary rToF patient with RV dilation and dysfunction but rather abnormal if the same patient was well-trained. Moreover, it would be essential to conduct further studies, assigning specific physical training protocols to rToF patients appropriate for their health condition, and assess them with MRI. This would allow us to unquestionably evaluate the effective improvement in oxygen consumption with a tailored physical exercise protocol on each patient group divided by RV dilation (e.g., RVEDVi < or >140 mL/m^2^) and/or RV dysfunction (e.g., RVEF < or >47%). Such findings would help us to accurately understand the correct type of exercise training to prescribe for each patient to optimize their long-term follow-up. This is because we believe that their behavior in everyday life (regular physical activity and nutrition appropriate to their cardiac condition) is fundamental for obtaining the best results and not just the regular medical checkups they undergo.

Lastly, a longitudinal study on serial changes in CPET parameters, MRI findings and patients’ physical activity levels will allow us to take a huge step forward in determining the optimal timing for PVR.

### 5.4. Ventricular Arrhythmias in the rToF Population

In our cohort, VT was noted in a few patients (8.4%), who presented with LV systolic dysfunction (although no one showed an LVEF <50%), older age, and a lower peak HR reached during CPET (which is typically influenced by age). Considering that we dealt with a population affected by known cardiac issues, it is important to stress that a link between ventricular ectopic beats and age has already been established. Hence, it is not possible to dismiss the fact that these arrhythmic occurrences were at least partially related to the increasing age [58].

### 5.5. Study Limitations

The present study has only included patients with a substantially preserved RVEF, as none had a dysfunction below 45%. Therefore, the lack of correlation between CPET parameters and RV dysfunction could be attributed to this fact. Similarly, only a few patients in our series (5 out of 83, 6%) showed a severe RV dilation (i.e., RVEDVi > 160 mL/m^2^). A deeper exploration of this subgroup of patients (severely dilated with at least mildly reduced systolic function in NYHA class I) would probably give us more valuable information for an even more accurate prognostic stratification. A small number of patients did not perform a maximal CPET (8, 9.5%), which could have minimally affected the results. Lastly, our cohort’s limited number of patients could be responsible for the lack of a significant correlation between some MRI and CPET parameters.

### 5.6. Conclusions

A reduction in aerobic capacity was observed in our cohort of rToF patients with preserved biventricular function and NYHA class I. The IPAQ (subjective data reported by the patient) associated with CPET (objective data evaluated by the operator) could provide a general picture of the patient’s condition. In addition to peak VO_2_, OUES (a submaximal index of exercise tolerance) revealed a strong correlation with exercise performance. Therefore, this parameter could be useful in this population whenever maximal exercise testing is not achievable. The degree of pulmonary insufficiency and severe RV dilation, in the presence of a preserved biventricular function/mild EF reduction, seems to have no importance as long as the compensatory mechanism maintains an adequate stroke volume for a satisfactory functional capacity (even if not normal, when compared with a healthy individual). Currently, there are still no reliable data in the literature that show at which cut-off values of severe RV dilatation and right and/or left ventricular dysfunction this compensation mechanism begins to fail. Therefore, further research is required, including a greater number of patients with RV dilation >160 mL/m^2^ and with various degrees of right and/or left ventricular dysfunction to resolve this question, which is crucial for determining the best timing for PVR. Our work has led us to conclude that CPET is an indispensable tool in the rToF population, although, at times, some parameters are difficult to interpret in the individual patient. Regular physical activity is confirmed to be correlated with better performance concerning both the peak VO_2_/Kg. Therefore, a physical activity intervention tailored for each rToF patient could also improve the long-term prognosis in this population.

## Figures and Tables

**Table 1:** Characteristics of 84 rToF patients. Data are expressed as median values and interquartile ranges for continuous variables and as counts and percentages for categorical variables.

Overall (*N* = 84)		
**Demographic features**	Male sex, n (%)	41 (48.8%)
	Age at repair (months) (median, IQR)	13.4 (0.0–87.0)
	Age at CPET (yrs) (median, IQR)	21.1 (15.0–30.0)
	Time between surgery and CPET (yrs) (median, IQR)	20.2 (10.2–28.9)
	BSA (m^2^) (median, IQR)	1.7 (1.1–2.2)
	BMI (median, IQR)	22.6 (15.6–33.3)
**MRI parameters**	RVEDV (mL) (median, IQR)	205.0 (106.0–327.9)
	RVEDVi (mL/m^2^) (median, IQR)	122.1 (63.3–174.3)
	RVESV (mL) (median, IQR)	92.2 (43.0–152.0)
	RVESVi (mL/m^2^) (median, IQR)	54.9 (25.7–84.6)
	RVEF (%) (median, IQR)	55.0 (45–69.0)
	RVSV (mL/beat) (median, IQR)	109.7 (6.0–182.5)
	LVEDV (mL) (median, IQR)	125.0 (69.0–206.5)
	LVEDVi (mL/m^2^) (median, IQR)	75.4 (48.8–113.7)
	LVESV (mL) (median, IQR)	54.1 (27.0–86.0)
	LVEDVi (mL/m^2^) (median, IQR)	33.5 (19.9–52.9)
	LVEF, (%) (median, IQR)	57.0 (45.5–66.0)
	LVSV (mL/beat) (median, IQR)	69.8 (42.0–108.3)
	PR (%) (median, IQR)	37.4 (27.0–60.0)
	RVOTO and/or PAs branches stenosis, n (%)	8 (9.5)
**CPET values**	Total test duration (s) (median, IQR)	607 (551–724)
	Peak HR (bpm) (median, IQR)	176 (167–182)
	Peak HR (%) (median, IQR)	88 (85–92)
	HR at AT (bpm) (median, IQR)	128 (119–138)
	Peak VO_2_ (mL/min) (median, IQR)	1885 (1530–2166)
	Peak VO_2_/Kg (mL/min/Kg) (median, IQR)	31 (25–33)
	Peak VO_2_/Kg (% of predicted) (median, IQR)	68 (61–78)
	VO_2_ at AT (mL/min/Kg) (median, IQR)	19.7 (18.3–23.7)
	Peak RER (median, IQR)	1.09 (1.02–1.15)
	Peak O_2_ pulse (mL/bpm) (median, IQR)	10.4 (8.81; 12.28)
	Peak O_2_ pulse (% of predicted) (median, IQR)	81 (71; 93)
	O_2_ pulse trend n (%)	
	− Increasing − Flattening − Decline	7 (10.3%) 58 (85.3%) 3 (4.4%)
	OUES (mL/min/L/min) (median, IQR)	1908 (1665–2538)
	OUES (% of predicted) (median, IQR)	79 (68–85)
	VE/VCO_2_ at AT (median, IQR)	27.5 (25.0–29.9)
	VE/VCO_2_ slope (AT) (median, IQR)	25.4 (23.6–28.9)
	Peak VE/VCO_2_ (median, IQR)	31.1 (27.3–34.3)
	VE/VCO_2_ slope (VCP) (median, IQR)	28.3 (25.5–31.5)
	VE/VCO_2_ slope (stop) (median, IQR)	30.7 (27.5–34.5)
	FVC (l) (median, IQR)	3.6 (2.97–4.26)
	FEV1 (l) (median, IQR)	3.1 (2.77–3.79)
	Peak VE (l/min) (median, IQR)	61 (54–76)
	Peak VE/VO_2_ (median, IQR)	33.0 (28.9–38.2)
	BR (%) (median, IQR)	50 (40–57)

Legend: AT: anaerobic threshold; BR: breathing reserve. BSA: Body surface area; HR: heart rate; LVEDV: left ventricular end-diastolic volume; LVEDVi: left ventricular end-diastolic volume indexed to body surface area; LVEF: left ventricular ejection fraction; LVESV: left ventricular end-systolic value; LVESVi: left ventricular end-systolic volume indexed to body surface area; LVSV: left ventricular stroke volume; OUES: oxygen uptake efficiency slope; PR: pulmonary regurgitation; RER: respiratory exchange ratio; RVEDV: right ventricular end-diastolic volume; RVEDVi: right ventricular end-diastolic volume indexed to body surface area; RVEF: right ventricular ejection fraction; RVESV: right ventricular end-systolic volume; RVESVi: right ventricular end-systolic volume indexed to body surface area; RVSV: right ventricular stroke volume; VE/VCO_2_: ventilatory equivalent for CO_2_; VE: ventilation; VO_2_: oxygen consumption.

**Table 2:** Comparison between rToF patients with normal right ventricular ejection fraction (RVEF) and rToF patients with reduced RVEF for clinical, cardiac magnetic resonance (CMR), and cardiopulmonary exercise testing (CPET) parameters.

	RVEF ≤ 51%	RVEF > 51%	*p* Value
** *n***	28	56	
** age at CPET (mean (SD))**	26.14 (8.85)	21.01 (7.61)	**0.007**
** IPAQ (%)**			0.303
** 0**	6 (20.7)	20 (36.4)	
** 1**	21 (72.4)	33 (60.0)	
** 2**	2 (6.9)	2 (3.6)	
** male (%)**	16 (55.2)	25 (45.5)	0.537
** BMI (mean (SD))**	22.00 (2.41)	22.82 (4.03)	0.321
** RVEDVi (mean (SD))**	128.50 (21.21)	121.46 (21.48)	0.155
** RVESVi (mean (SD))**	65.23 (11.18)	51.22 (11.43)	**<0.001**
** LVSV (mean (SD))**	73.05 (16.27)	72.66 (14.50)	0.913
** RVSV (mean (SD))**	106.45 (19.02)	116.94 (26.13)	0.061
** PR (mean (SD))**	31.97 (17.45)	34.45 (13.06)	0.463
** Peak RER (mean (SD))**	1.07 (0.10)	1.10 (0.10)	0.160
** VE/VCO_2_ slope (stop) (mean (SD))**	30.74 (4.69)	30.68 (5.13)	0.958
** VE/VCO_2_ slope (VCP) (mean (SD))**	28.52 (3.99)	28.94 (4.74)	0.724
** peak VE/VCO_2_ (mean (SD))**	31.01 (4.50)	31.32 (5.77)	0.806
** VE/VCO_2_ slope (AT) (mean (SD))**	25.75 (3.47)	26.73 (4.44)	0.316
** VE/VCO_2_ AT (mean (SD))**	27.39 (3.47)	28.55 (4.68)	0.258
** OUES (mean (SD))**	2107.36 (579.95)	2083.61 (617.28)	0.869
** Trend VO_2_ HR (mean (SD))**	1.96 (0.36)	1.93 (0.39)	0.796
** Peak oxygen pulse (mean (SD))**	11.26 (2.64)	10.46 (3.20)	0.249
** VO_2_ AT (mean (SD))**	21.00 (4.23)	21.14 (4.41)	0.889
** Peak VO_2_/kg (mean (SD))**	29.61 (7.11)	30.42 (6.87)	0.612
** Peak VO_2_ (mean (SD))**	1896.24 (458.10)	1884.59 (522.23)	0.920

Legend: IPAQ: international physical activity questionnaire; BMI: body mass index; AT: anaerobic threshold; RVEDVi: right ventricular end-diastolic volume normalized for body surface area; RVESVi: right ventricular end-systolic volume normalized for body surface area; LVSV: left ventricular stroke volume; RVSV: right ventricular stroke volume; PR: pulmonary regurgitation; RER: respiratory exchange ratio; VE/VCO_2_: ventilatory equivalent for CO_2_; OUES: oxygen uptake efficiency slope; VO_2_: oxygen consumption; HR: heart rate.

## Data Availability

The data presented in this study are available on request from the corresponding author.
